# Artificial intelligence in healthcare: an Italian perspective on ethical and medico-legal implications

**DOI:** 10.3389/fmed.2024.1343456

**Published:** 2024-06-03

**Authors:** Sara Sablone, Mara Bellino, Andrea Nicola Cardinale, Massimiliano Esposito, Francesco Sessa, Monica Salerno

**Affiliations:** ^1^Section of Legal Medicine, Interdisciplinary Department of Medicine, Bari Policlinico Hospital, University of Bari Aldo Moro, Bari, Italy; ^2^Faculty of Medicine and Surgery, Kore University of Enna, Enna, Italy; ^3^Department of Medical, Surgical and Advanced Technologies “G.F. Ingrassia”, University of Catania, Catania, Italy

**Keywords:** artificial intelligence, medico-legal practice, decision-making process, medical professional liability, black box, informed consent, ethics

## Abstract

Artificial intelligence (AI) is a multidisciplinary field intersecting computer science, cognitive science, and other disciplines, able to address the creation of systems that perform tasks generally requiring human intelligence. It consists of algorithms and computational methods that allow machines to learn from data, make decisions, and perform complex tasks, aiming to develop an intelligent system that can work independently or collaboratively with humans. Since AI technologies may help physicians in life-threatening disease prevention and diagnosis and make treatment smart and more targeted, they are spreading in health services. Indeed, humans and machines have unique strengths and weaknesses and can complement each other in providing and optimizing healthcare. However, the healthcare implementation of these technologies is related to emerging ethical and deontological issues regarding the fearsome reduction of doctors’ decision-making autonomy and acting discretion, generally strongly conditioned by cognitive elements concerning the specific clinical case. Moreover, this new operational dimension also modifies the usual allocation system of responsibilities in case of adverse events due to healthcare malpractice, thus probably imposing a redefinition of the established medico-legal assessment criteria of medical professional liability. This article outlines the new challenges arising from AI healthcare integration and the possible ways to overcome them, with a focus on Italian legal framework. In this evolving and transitional context emerges the need to balance the human dimension with the artificial one, without mutual exclusion, for a new concept of medicine “with” machines and not “of” machines.

## 1 Introduction

Artificial intelligence (AI) is a multidisciplinary field addressing the creation of systems that perform tasks generally requiring human intelligence. It encompasses a much broader spectrum than the mere imitation of human intelligence through information and communication technologies since it regards the development of algorithms and computational methods that enable machines to correctly interpret external data, learn from them, and use those learnings to achieve specific goals and tasks through flexible adaptation (1). There are two different types of AI: weak AI, designed to perform a narrow task (i.e., facial recognition, Internet Siri search, self-driving car, etc.), and strong AI, which is the speculative intelligence of a machine able to understand or learn any intelligent task, thus assisting human in unraveling the faced problem (2–4).

Nowadays, AI is undergoing widespread use among healthcare services. In particular, AI can assist physicians in preventing and classifying the patient’s conditions by reducing diagnostic and pathophysiological uncertainty. Moreover, AI helps in considering which treatment will be most appropriate for the patient, reducing prognostic uncertainty and increasing the prediction of the onset or evolution of pathologies. In addition, AI is currently used to support healthcare workers by providing the most updated and appropriate guidelines, which can be consulted even while working on the ward. AI could also improve and make workflows more efficient and smoother within healthcare facilities, from emergency management to the interpretation of images, but also in clinical trials, precision medicine (medical robots), and the pharmaceutical sector (5).

Unavoidably, AI integration in healthcare requires AI technologies to be embedded into the workflows to support clinical decision-making at the point of care and reduce human errors related to fatigue (6). In turn, human oversight remains essential and necessary to design, program, and operate AI, thus preventing any unforeseen errors from occurring (4). Nevertheless, because of this necessary coexistence in the decision-making sphere, medico-legal issues regarding medical liability arise. Primarily, it is crucial to determine whether AI medical technologies should be classified solely as tools or if they merit some degree of legal subjectivity (i.e., “electronic personality”) (7, 8). Subsequently, it is vital to evaluate the legal impact of AI systems in physicians’ decision-making process. From these two issues arise the topic regarding ethics and professional liability by applying AI to medical practice. The main discussion about ethics deals with the legal qualification of the healthcare professional for patient damages deriving, for example, from a treatment prescription developed by an algorithm. Further questions concern the liability of physicians recurring when the machine decision-making is incomprehensible and opaque. Hence, the primary challenge currently lies in comprehending the nuanced aspects of liability associated with AI medical device utilization among the involved stakeholders, such as physicians, healthcare institutions, medical device manufacturers, and the devices themselves (9).

In this scenario, by a thorough examination of the Italian legislative system and comparison thereof with the international legal framework, this article aims to highlight the new challenges arising from AI healthcare integration and the possible ways to overcome them.

## 2 AI in medico-legal practice: ethics and liability implications

### 2.1 AI in medico-legal practice

The healthcare legal landscape worldwide is intricate and multifaceted, with different regulations at national and international levels. As AI technology increasingly permeates the healthcare sector and holds the promise of transforming it, concerns about its impact on patient safety, doctor-patient relationship, and medical liability have grown (10). The potential for unintended consequences at a larger scale, such as within state-wide or national health systems, has led to the cautious adoption of AI in everyday healthcare (11). Addressing the robustness, interpretability, and accountability of AI is crucial before widespread implementation (12, 13). The concept of ‘responsible AI’ has gained global attention, with different international publications focusing on values-based principles to promote trustworthy AI (14). Particularly relevant are the principles of accountability, transparency, and explainability, which play a pivotal role in determining liability in case of medical errors caused by AI technology. Moreover, the literature emphasizes the significance of regulations to ensure product safety and minimum safety standards upheld in AI applications within the medical domain. However, the inherent complexity and the ‘black box’ nature of the AI decision-making process pose significant challenges in assigning liability under traditional civil wrong paradigms (15).

### 2.2 Ethics

AI in medicine represents a new opportunity to enhance healthcare, albeit with the potential to introduce rising risks by reshaping the role of physicians in clinical practice (16). Nowadays, AI use first requires an accurate collection and selection of data on which the AI algorithm will work. This process should be assessed by programmers and technicians using machines, but the obtained algorithm could lack transparency. Indeed, programmers do not always know how the machine achieved certain results (the so-called “black-box problem”). In simple terms, machine automation can bring “opacity” to the algorithm, making it impossible to trace all the information on how to reach a conclusion or suggest a decision. A human being cannot analyze the enormous amount of calculations made by the algorithm and find out exactly how the machine works to decide. Thus, this opacity increases the risk that health practitioners cannot validate the AI proposal in an attempt to make their own decisions (5). As a result, omitting explainability in clinical decision support systems threatens core ethical values (17). Indeed, legal uncertainty occurs when AI is asked to perform tasks with greater independence from physicians or when physicians blindly rely on AI algorithms that may be unverifiable (18). With this background, a medicine “with” machines, and not “of” machines, appears to be impossible by now, as there is no way for physician to overview the decision-making process elaborated by AI. In this context, to avoid AI systems from overpowering humans in the healthcare decision-making process, the European Court of Human Rights declares that each State has to safeguard individual freedom against potential interference from third parties. It implies a state control of AI applications in health systems to guarantee the autonomy of human decisions (19).

Present-day medicine is evidence-based and aimed to address healthcare practitioners’ interventions and actions to make them adherent to clinical guidelines (20). Guidelines and good clinical practice recommendations supplied by Evidence-Based Medicine (EBM) are provided by scientific societies and associations after clinical and pre-clinical research (21). Hence, it is important to emphasize the distinctions between EBM methodology and the application of AI in the medical field. Both are namely aimed to address clinical management to obtain better outcomes and predictions, thus narrowing the imprecision of medicine making the latter more “scientific”. Anyway, there is a huge gap between the validation of these tools: due to the “black-box” nature of AI hindering the data elaboration process, the outcome of the algorithm may not be evaluated by the physician utilizing them. As a result, the physician might not fully know the reasons behind a medical decision. On the contrary, guidelines and recommendations of EBM are the outcome of different high-quality studies and research, motivated by the scientific and logical process, thus intelligibly evaluable by the physician recurring to them. In this way, the healthcare practitioner can differentiate whether guidelines are consistent with the specificity of the case. Another time, the main hurdle to overcome is the AI machines’ lack of transparency, which makes it impossible for users to understand the reasoning behind the result, making them not self-sufficient in healthcare management.

Regarding specific population historical datasets, the Food and Drug Administration (FDA) strives to address bias that may arise during the training or utilization of AI algorithms due to historical data and human prejudices. Bias and fairness play a crucial role in establishing trust. Reducing bias in AI requires the implementation of fairness protocols, regular audits’ conduction, and several viewpoints’ incorporation throughout the AI development process. A current topical focus on bias mitigating and trust increasing advocates the inclusion of human-in-the-loop (human-centered AI). By means such as counterfactual explanations, it could address bias and boost trust, allowing individuals to better understand unfamiliar processes and explore hypothetical input scenarios that affect outcomes (22).

In Italy, the current normative reference is Directive 85/374/EEC based on the Original Decree of the President of the Republic of 24 May 1988, n. 224, today transfused in art. 114-127 of Legislative Decree no. 206 of 6 September 2005 (23). This Legislative Decree issued the Consumer Code to ensure the protection of consumers and users, but also the harmonization and reorganization of the regulations concerning the purchasing and consumption processes, in compliance with European principles (24).

In Italy, the Law n. 219 of 2017 is also currently in force (25). It regulates the aspects related to patient’s informed consent to specific diagnostic and therapeutic interventions, including those provided with the help of AI medical devices. In particular, this law protects the patient’s right to be fully informed about the diagnosis, prognosis, benefits, and risks of diagnostic tests and medical treatments and their possible alternatives [“Norme in materia di consenso informato e di disposizioni anticipate di trattamento (Legge 219/2017)”, 2017]. Therefore, it is advisable to inform patients about the “opaque aspects” of AI technology besides highlighting the benefits these tools can offer, thus letting them decide whether to employ AI assistance. This regulatory guidance is paramount since informed consent obtainedfor AI device employment is a prerequisite for potential professional liability assessment.

### 2.3 Liability

AI in healthcare may be defined as the declination of intelligent behaviors characterizing all medical activities.

AI in healthcare encompasses all areas where medical knowledge needs to be represented and extended through different types of reasoning. On the other hand, AI considers intelligent behaviors, which are the basis of the many decision-making activities in medicine, such as diagnosis, therapy, prognosis, and patient monitoring management. These activities characterize clinical practice and include the ability to merge and use basic knowledge, patient-specific knowledge, and environment tools to make the best possible decision regarding the evolution of the patient’s health status (or of entire patient groups) within an acceptable time frame.

To avoid medical malpractice liability, physicians must provide healthcare by considering available resources (26). However, the situation becomes more complicated when an AI device is involved because there is no case law on liability for AI systems employment yet. Current law shields physicians from liability as long as they follow the standard of care, the “safest” way to use medical AI, but this approach incentivizes physicians to minimize the potential power of AI (18, 27). Indeed, the threat of liability encourages physicians to the standard of care, with the possibility to reject AI recommendations, in some cases to patients’ detriment.

New concerns have emerged surrounding the potential recognition of artificial intelligence as a distinct legal entity due to its autonomous decision-making skills. Researchers hold differing views on this issue. Bottomley and Thaldar highlight inherent limitations. Specifically, acknowledging AI as a legal entity would impact medical-legal disputes since it provides a tool to complainants who could gather evidence from AI systems by examining it as a witness. However, this advantage could be restricted as many modern systems lack transparent reasoning. Moreover, the main disparity between human and AI decision-making lies in the morality of their actions (28). Indeed, human decision-making is influenced by moral considerations, a dimension completely absent in computer systems (2). Within this context, different authors have explored the “principle-agent relationship”. Specifically, when assigning responsibility for medical acts to AI or the healthcare provider who strictly follows the algorithm instructions, a system grounded in the human-in-the-loop model seems more fitting. It entails humans acting as overseers of AI recommendations before their application in healthcare settings. The challenge arises when AI computational power far exceeds human intellect. In such instances, if AI is not embedded into care standards, care providers must take the risk by choosing between adherence to recognized guidelines or relying on AI outputs (28). The lack of a clear definition of the responsibility of both AI and the physician who uses it further complicates the ability to assess fault-based liability, due to the ambiguity surrounding carelessness (28). An alternative to fault-based liability is represented by strict liability (also known as no-fault liability), which simplifies the burden for claimants. Indeed, it becomes sufficient to demonstrate the harm rather than proving the existence of fault. However, this system is generally applied in cases of unforeseeable events to date: the definition of harm from AI as an unforeseeable event remains uncertain. A further limitation is the economic impact that such a measure would entail, as many litigations would result in compensations even in the burden-of-proof absence.

Some normative steps have been made to overcome these medico-legal issues. Indeed, in recent years the AI sector has been the subject of a series of interventions by the European Community aimed at outlining a possible common framework of reference to align the individual disciplines of the Member States (23, 29). The European legal basis lies in Article 114 of the Treaty on the Functioning of the European Union (TFEU), which sets rules regarding the use of products and services making use of AI technologies and providing stand-alone AI systems. Some Member States are already considering national rules to ensure that AI is safe and is developed and used in compliance with fundamental rights obligations. On 21st April 2021, the European Commission promulgated the Artificial Intelligence Act, a specific framework aimed at providing legal certainty and proposing a new liability regime in the AI integration context (19, 30). This regime is based on existing legal principles and adopts a risk-based scheme distinguishing high-risk AI systems from low-risk AI systems (31). The former comprehends medical AI devices which are consequently subjected to strict liability. The AI Act proposes to evade the “black-box problem” by extending liability to individuals involved in the creation, maintenance, or control of AI systems (32). The legislative solutions contained in this Act will have to be approved by the Member States and the European Parliament and their entry into force will not take place before the current year. The FDA also promotes the safe use of AI in healthcare through an action plan to maintain oversight of AI as a medical device. Moreover, the FDA aims to allow traceability and increase transparency by requiring programmers to precisely describe the functioning of their AI devices (29, 33).

For what concerns the allocation of the medical civil liability for damages resulting from the use of AI technologies, it will follow a double track. On one hand, the new frontiers of legal mediation will lead to the extension of liability to the medical devices’ manufacturers, programmers, and trainers, for harmful behavior resulting from malfunctioning of the algorithm. This liability extension should be carried out according to the Directives 2006/42/EC (All. XI) (34) and 2001/95/EC on safety machines and products in general (35). On the other hand, the question is whether it correct to point out the responsibility of the device end users, i.e., the healthcare professionals. According to Article 7 of the Italian Gelli-Bianco Law (8th March 2017 no. 24) (36–42), in case of patient death or personal injuries occurring in the healthcare delivery, the physician causing it due to incompetence and inexperience is not liable if guidelines or good clinical practice have been observed, provided they apply to the specific case. Since the result implementation of AI’s decision-making algorithm is far different from the observation of EBM guidelines, the principles of the attribution of liability need revision. There is no cognition of single steps leading to the final decision, thus it is impossible to identify a “juridical person” as an active actor in the decision-making process in AI application in medicine. Hence, there is a need for a new legal regulation in matters of medical professional liability in the case of AI applications. The target would be to promote a conscious use of this new technological tool and an active “cooperation” of the machine with healthcare providers, whilst guaranteeing the ethical-legal safeguarding of patients during healthcare delivery.

The pertinent literature underscores the lack of clear and unified legislation concerning the liability of healthcare professionals employing AI.

In particular, Atabekov (43) («АНАЛИЗ ПОДХОДОВ ОПРЕДЕЛЕНИЯ ЮРИДИЧЕСКОЙ ОТВЕТСТВЕННОСТИ ЗА ДЕЙСТВИЯ ИСКУССТВЕННОГО ИНТЕЛЛЕКТА В МЕДИЦИНСКОЙ СΦЕРЕ: ОПЫТ США И РОССИИ. – тема научной статьи по праву читайте бесплатно текст научно-исследовательской работы в электронной библиотеке Киберенинка», s.d.) highlights that in Russia AI is considered a high-risk medical device (according to the decree of the Ministry of Health of the Russian Federation No. 4 of 06/06/2012) and, as such, is subject to state registration (decree of the Government of the Russian Federation No. 323 of 12/27/2011). However, universal rules for the distribution of medical liability in case of harm to patients are not available. Hu and Yuan (44) stress the lack of a dedicated and organized legal framework concerning healthcare liability stemming from AI utilization in China, advocating for the adoption of regulations inspired by those established in the European Union. About the African continent, Townsend et al. (45) undertook a comprehensive investigation spanning 12 African countries, aiming to delineate the liability profiles of healthcare sector AI product providers. Although Townsend et al. (45) associate the immaturity of AI applications in healthcare systems with the economic status of African countries, our literature review underscores that AI legislation remains immature on a global scale.

## 3 Discussion and conclusion

The beneficial influence of AI systems is exponentially increasing in all medical fields. Indeed, AI systems are significantly changing the clinical practice, the physician’s decision-making methods, and the doctor-patient relationship. This evolution is allowed by the huge amount of digital data available, but also by the most recent technological developments in computational power, storage capacity, and engineering innovation in AI methods and tools. However, this technology is not free from risks, thus the necessity to properly overcome them through the building of trustworthy AI systems. For this reason, AI systems require rigorous scientific validation and should adhere to laws and regulations, but also to ethical principles and values. Firstly, strict rules for the approval and registration of AI systems are needed. Moreover, it should be characterized by technical and social robustness to ensure safety in the healthcare system (23, 29). In particular, this could be achieved through the establishment of a computerized infrastructure aimed at managing data (23). It is also vital to establish a standard practice by the provision of guiding principles regarding the use and application of AI, but also the acquisition of informed consent by patients. Moreover, proactive leadership from professional associations is needed to increase public confidence in the safety and efficacy of medical AI devices and to allow future innovation in this auspicious area (18). It may only become feasible when AI technologies are developed to enhance transparency in their calculation process. In this context, there is a need for urgent intervention in the regulatory framework to address the inadequacy of current regulations regarding AI’s liability and the associated risks.

In the European context, it seems appropriate to proceed as soon as possible, establishing a separation between policy aspects and liability profiles (46). The formers can be defined at the European level, promoting international cooperation for reliable and sustainable AI. The latter must be defined by the various legal systems because of the difficult achievement of homogeneity in medical malpractice regulation. For example, in Italy healthcare professionals continue to follow the Gelli-Bianco Law dictates, which provide the assumption of professional liability also in the case of AI medical device employment. Anyway, it would be necessary to update this system bearing in mind that the failure of treatment and the worsening of the patient’s health following the use of AI do not necessarily imply the existence of a medical error. In other words, the use of emerging technologies always requires the healthcare provider to act with prudence, diligence, and expertise according to the needs of the specific circumstances. But if the unfortunate event is dependent on technological instruments that are not intelligible, liability should be taken by the manufacturer or by the programmer (9).

Ultimately, the rapid advancement of AI technologies is not globally matched by a legislative framework evolution, as emphasized by several authors (8, 47). To harmonize the human and AI decision-making process, it would be appropriate to develop a supervisory mechanism regulating the product lifecycle. As argued by Terry (47), audits on the algorithm should be conducted by specialized figures such as “super-regulators.” This could overcome the ethical issues related to the “morality” of artificial intelligence since the super-regulator could perform quality checks on the input data of artificial intelligence. Another issue concerns the professional liability attribution system in case of claims when AI is used. In this context, a strict liability system could be adopted. Due to the significant economic investment required by hospitals to sustain such a system, a mandatory insurance system with a guarantee fund could be established. Naidoo et al. (8) suggest replacing the typical “Western” fault-based legal liability with a “reconciliatory” approach. In this perspective, Healthcare Reconciliation Commissions should promote audits aiming to address questions about who acted and what failed in the process to develop guidelines and good practices for AI’s application in healthcare systems. This approach could be of utmost importance due to the actual immaturity of the integration of AI algorithms in health systems.

In conclusion, it is crucial to follow the transition of the healthcare system due to the emerging AI medical devices, paying peculiar attention to critical aspects of the relationship between medicine and law. Indeed, a collaboration between law and sciences through a mutual cognitive exchange is required to assess new responsibilities, new rules, and new evolutionary interpretations, especially regarding the AI-assisted decision-making process and professional liability.

## Author contributions

SS: Conceptualization, Funding acquisition, Investigation, Methodology, Project administration, Resources, Supervision, Validation, Visualization, Writing – original draft, Writing – review and editing. MB: Data curation, Investigation, Writing – original draft. AC: Data curation, Investigation, Writing – original draft. ME: Formal analysis, Methodology, Validation, Writing – review and editing. FS: Formal analysis, Methodology, Validation, Visualization, Writing – review and editing. MS: Methodology, Project administration, Supervision, Validation, Visualization, Writing – review and editing.
